# Fluoroquinolone Exposure and Cancer Risk in Interstitial Lung Disease: A Propensity-Score-Matched Cohort Study Using Cox and Competing-Risk Models

**DOI:** 10.3390/ph19071067

**Published:** 2026-07-10

**Authors:** Yi-Fan Sun, Yu-Ting Chiu, Yung-En Ko, Yu-Wei Huang, Liang-Kai Hsieh, Cheng-Li Lin, Chia-Hung Kao, Jun-Jun Yeh

**Affiliations:** 1 Department of Family Medicine, Geriatric Medicine, Chest Medicine and Medical Research, Ditmanson Medical Foundation Chia-Yi Christian Hospital, No. 539, Zhongxiao Rd., East Dist., Chiayi 600566, Taiwan; stevensunyifan85@gmail.com (Y.-F.S.); 05442@cych.org.tw (Y.-T.C.); yungenko1027@gmail.com (Y.-E.K.); a10263908@gmail.com (L.-K.H.); 2 Department of Laboratory Medicine, Ditmanson Medical Foundation Chia-Yi Christian Hospital, Chiayi 600566, Taiwan; 3 College of Medicine, China Medical University, Taichung 40402, Taiwan; orangechengli@gmail.com; 4 Management Office for Health Data, China Medical University Hospital, Taichung 40402, Taiwan; 5 Artificial Intelligence and Robotics Innovation Center, China Medical University Hospital, Taichung 40402, Taiwan; 6 Department of Nuclear Medicine and PET Center, China Medical University Hospital, Taichung 40402, Taiwan; 7 Graduate Institute of Biomedical Sciences, School of Medicine, College of Medicine, China Medical University, No. 2, Yuh-Der Road, Taichung 40402, Taiwan; 8 Department of Bioinformatics and Medical Engineering, Asia University, Taichung 41354, Taiwan

**Keywords:** fluoroquinolones, cancer risk, interstitial lung disease, immortal time bias, propensity score matching, competing-risk analysis, confounding by indication, reverse causation, pharmacoepidemiology

## Abstract

**Background:** This study aimed to comprehensively investigate the complex association between the use of fluoroquinolone (FQ) antibiotics and cancer risk, with a specific focus on patients with interstitial lung disease (ILD)—a unique clinical population characterized by a high inflammatory burden and a high susceptibility to infections. **Methods:** We conducted a large-scale retrospective cohort study using a high-quality clinical database. A total of 7906 matched patients (3953 pairs) were included after propensity score matching (PSM). Three complementary statistical models were applied: the standard Cox proportional hazards model, the time-dependent Cox regression model, and the Fine–Gray competing-risks model, to provide a multidimensional assessment of cancer risk. **Results:** A total of 7906 matched patients (3953 pairs) were followed. After strictly defining the index date to eliminate immortal time bias, FQ exposure was associated with an increased risk of all-cause cancer in the standard Cox model (adjusted HR 1.45; 95% CI, 1.20–1.76) and the competing risk model (adjusted SHR 1.28; 95% CI, 1.06–1.55). Site-specific analyses revealed elevated risks for certain malignancies, notably prostate cancer. Importantly, when modeled as a continuous variable, the cumulative dose of fluoroquinolones showed no significant dose–response relationship with overall cancer risk (adjusted HR 0.99; 95% CI, 0.99–1.00). **Conclusions:** After correcting for immortal time bias, the previously hypothesized protective effect of fluoroquinolones on cancer risk was not observed. The increased risk observed in categorical models, coupled with a lack of a continuous dose–response, strongly suggests that these findings are driven by confounding by indication and reverse causation (i.e., frequent infections masking undiagnosed malignancies or reflecting severe underlying ILD), rather than a direct pharmacological effect.

## 1. Introduction

### 1.1. Research Background and Rationale

Since their introduction in the 1980s, fluoroquinolone (FQ) antibiotics have become a cornerstone of global clinical practice due to their broad-spectrum antimicrobial activity, excellent tissue penetration, and high oral bioavailability [1]. They are widely prescribed for respiratory, urinary tract, and intra-abdominal infections [1]. However, with their exponential increase in use, concerns regarding their long-term safety have intensified. Beyond well-documented adverse effects such as tendinopathy, aortic aneurysm, and dysglycemia, whether FQs possess carcinogenic or anticarcinogenic properties has emerged as a major point of contention in pharmacoepidemiology [2,3,4,5].

From a molecular perspective, FQs exert their antibacterial effects by inhibiting bacterial DNA gyrase and topoisomerase IV, thereby disrupting DNA replication. Although their affinity for eukaryotic topoisomerases is substantially lower, high concentrations may induce DNA double-strand breaks, oxidative stress, and mitochondrial dysfunction in mammalian cells, theoretically conferring mutagenic and carcinogenic potential [2,3,6]. Conversely, chronic inflammation is widely recognized as a central driver of carcinogenesis. Beyond their antimicrobial effects, FQs have demonstrated immunomodulatory properties, including the suppression of pro-inflammatory cytokines (e.g., IL-6, TNF-α) and matrix metalloproteinase (MMP) activity [2,3,4,7]. These properties suggest that FQs may interrupt the “infection–inflammation–cancer” axis and exert chemopreventive effects [8,9].

### 1.2. The Unique Risk Profile of Patients with Interstitial Lung Disease

Patients with interstitial lung disease (ILD) represent a population of exceptional clinical and pathophysiological relevance. ILD comprises a heterogeneous group of disorders characterized by pulmonary interstitial fibrosis and chronic inflammatory cell infiltration [10]. These patients inherently face a markedly elevated risk of lung cancer—estimated to be 3–5 times higher than that of the general population [11]. Moreover, structural lung damage and the widespread use of immunosuppressive agents (e.g., corticosteroids, cyclophosphamide) predispose ILD patients to recurrent respiratory infections, resulting in substantially higher rates of FQ prescriptions compared with the general population [1,12]. This dual profile of “high baseline cancer risk” and “high drug exposure” renders the ILD cohort a natural experimental setting for evaluating the long-term carcinogenic or protective effects of FQs [11,12,13]. If FQs were truly carcinogenic, their effects would be amplified in the context of ILD-related genomic instability [13,14]. Conversely, if FQs effectively suppress infection-driven inflammatory cascades, their protective effects should be most evident in this population [4,7].

### 1.3. Study Objectives and Core Research Questions

Recognizing that prior observational studies have been heavily influenced by confounding by indication and detection bias, we employed propensity score matching to construct a highly homogeneous comparison cohort. This study aimed to address the following key scientific questions: (1) Overall effect: After adjusting for comorbidities and concomitant medications, does FQ use increase or decrease the overall cancer risk among patients with ILD? (2) Site-specific effects: Is there heterogeneity in cancer risk across different organ systems (e.g., lung, gastrointestinal tract, genitourinary system)? (3) Dose–response relationship: Do cumulative exposure duration and cumulative dose exhibit threshold or linear associations with cancer risk? (4) Methodological validation: How do standard Cox models, time-dependent models, and competing risk models differ in the context of high mortality among ILD patients, and which approach most closely reflects biological reality?

## 2. Results

**Figure 1:**
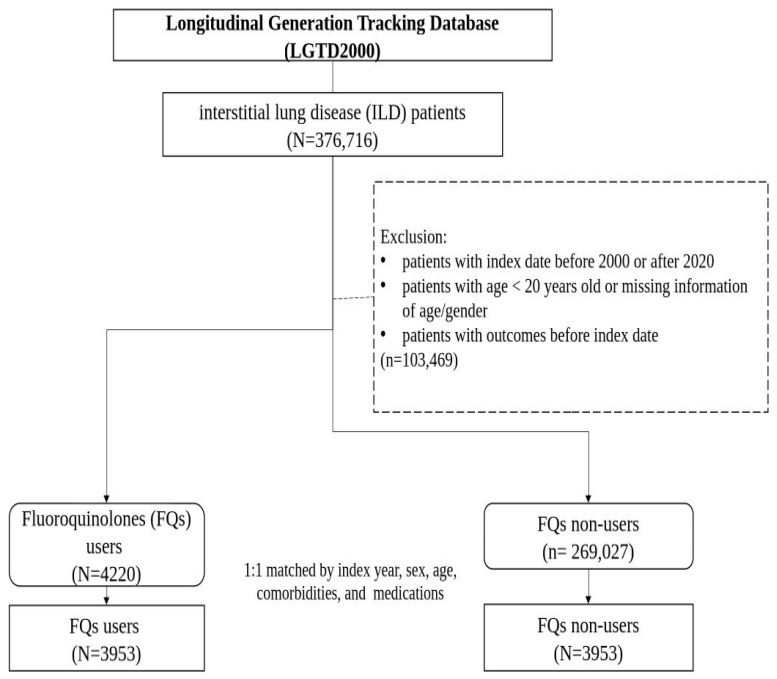
Study flow diagram for cohort construction and propensity score matching. Patients with interstitial lung disease (ILD) were identified from the database. After applying eligibility criteria (including exclusion of patients with prior cancer before the index date), patients were classified as fluoroquinolone (FQ) users or non-users. To avoid immortal time bias, the index date for FQ users was defined as the date of their first prescription, while non-users were assigned a randomized index date. Patients were then matched 1:1 using propensity scores based on demographics, comorbidities, and concomitant medications. The final propensity-score-matched cohort included 3953 FQ users and 3953 non-users (total N = 7906). Patients were followed from their respective index dates until incident cancer, death, or the end of the study period; the mean follow-up duration was 5.43 years for FQ users and 5.95 years for non-users.

**Figure 2:**
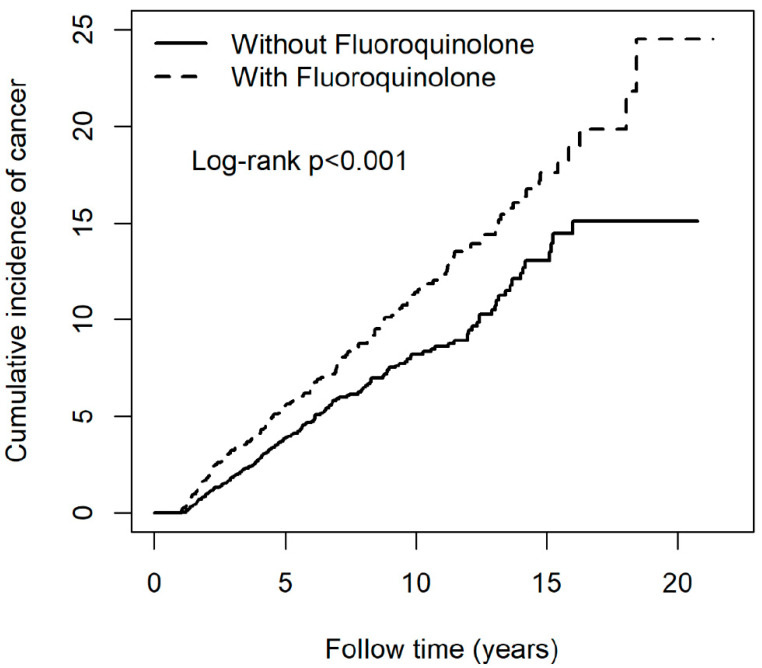
Kaplan–Meier cumulative incidence curves for overall cancer in fluoroquinolone (FQ) users and non-users after propensity score matching. The cumulative incidence of overall cancer was higher in FQ users than in non-users throughout follow-up. After rigorous index-date assignment to minimize immortal time bias, the Kaplan–Meier analysis demonstrated a statistically significant difference between the two groups (log-rank *p* < 0.001). These findings indicate that FQ exposure was associated with an increased long-term risk of cancer in patients with interstitial lung disease (ILD).

After propensity score matching and applying the rigorous index date assignment to avoid immortal time bias, 3953 patients were included in each cohort, yielding a total study population of 7906 individuals. The distributions of age, sex, baseline comorbidities, and concomitant medications were well balanced between fluoroquinolone (FQ) users and non-users (Table 1; Figure 1). The mean follow-up duration was 5.43 years for FQ users and 5.95 years for non-users. During follow-up, the cumulative incidence of cancer was higher among FQ users than among non-users, as illustrated by the Kaplan–Meier curves (Figure 2), and the difference between the two groups was statistically significant based on the log-rank test.

In the matched cohort, after accounting for immortal time bias, FQ use was associated with a higher risk of overall cancer compared with non-use. In the fully adjusted Cox proportional hazards model, the adjusted hazard ratio (aHR) for overall cancer among FQ users was 1.45 (95% CI, 1.20–1.76; *p* < 0.001) (Table 2).

Compared with non-users, FQ users had significantly higher risks of prostate cancer (aHR 5.50, 95% CI 2.25–13.48). Conversely, a lower risk was observed for immune-related cancers (aHR 0.62, 95% CI 0.44–0.87). No statistically significant associations were observed for several other cancer types, including lung, liver, and colorectal cancers (Table 3).

When stratified by cumulative duration of FQ therapy, no significant dose–response relationship or risk reduction was observed (Table 4). For colorectal cancer, the adjusted hazard ratios across all duration strata (≤35 days, 36–55 days, and >56 days) indicated non-significant increased risks (aHRs ranging from 1.01 to 1.53, all 95% CIs crossing 1.00). Similarly, for lung cancer, liver cancer, and immune-related cancers, the risks among FQ users were not significantly different from those of non-users, regardless of the cumulative exposure duration. These findings indicate that the previously hypothesized dose-dependent protective effects were not present after accounting for immortal time bias.

Analyses stratified by cumulative dose of FQs revealed no significant dose–response relationship (Table 5). Compared with non-users, patients receiving varying cumulative doses of FQs (≤3000 mg, 3001–6000 mg, and >6000 mg) did not exhibit any statistically significant reductions in cancer risk. For colorectal cancer, liver cancer, lung cancer, and immune-related cancers, the adjusted hazard ratios across all dose strata were not statistically significant, with all 95% confidence intervals crossing 1.00. These findings further corroborate that the previously observed dose-dependent protective associations were likely artifacts of immortal time bias, and that the cumulative dose of FQs does not directly modulate cancer risk in this cohort.

In the time-dependent Cox regression model, which treated FQ exposure as a time-varying variable, the association with an increased risk of cancer was further corroborated (Table 6). FQ exposure was associated with a significantly increased risk of overall cancer (aHR 2.07, 95% CI 1.57–2.72). Markedly elevated risks were observed for prostate cancer (aHR 9.95, 95% CI 4.75–20.8) and lung cancer (aHR 3.35, 95% CI 1.87–6.01), as well as for hematologic malignancies (aHR 4.09, 95% CI 1.51–11.1) and cancers of the bladder and kidney (aHR 3.72, 95% CI 1.55–8.93).

Because of the high mortality rate in patients with interstitial lung disease, a competing risk analysis using the Fine–Gray subdistribution hazard model was performed, treating death as a competing event (Table 7). In this model, consistent with the updated primary Cox analysis, FQ use remained associated with a significantly increased risk of overall cancer (adjusted subdistribution hazard ratio [aSHR] 1.28, 95% CI 1.06–1.55). However, no significant associations were observed for specific cancer subtypes, including colorectal cancer (aSHR 1.24, 95% CI 0.69–2.22), liver cancer (aSHR 0.88, 95% CI 0.53–1.47), lung cancer (aSHR 1.04, 95% CI 0.65–1.68), and immune-related cancers (aSHR 0.91, 95% CI 0.59–1.42).

### Continuous Exposure Variable Analysis

To robustly evaluate the dose–response relationship and address concerns regarding arbitrary exposure cutoffs, fluoroquinolone exposure was analyzed as a continuous variable (Table 8). In this model, the continuous cumulative dose of fluoroquinolones was not significantly associated with the risk of all-cause cancer (aHR 0.99, 95% CI 0.99–1.00). This lack of a continuous dose–response relationship further suggests that the significant associations observed in the categorical models may be driven by confounding factors rather than direct pharmacological effects.

## 3. Discussion

### 3.1. Main Findings

In this propensity-score–matched cohort study of patients with ILD, FQs exposure was associated with a higher long-term risk of overall cancer after rigorous index-date assignment to reduce immortal time bias. The association remained significant in the standard Cox model and was directionally consistent in the Fine–Gray competing-risk model and time-dependent Cox regression. These findings suggest that the previously observed protective association was not sustained after correction for immortal time bias [15,16,17,18]. Site-specific analyses showed marked heterogeneity. FQs exposure was associated with a substantially increased risk of prostate cancer, whereas immune-related cancers showed a reduced risk. In contrast, no consistent significant association was observed for several other major cancers, including lung, liver, and colorectal cancers. Importantly, cumulative duration- and dose-stratified analyses did not demonstrate a clear linear dose–response relationship. When cumulative dose was modeled as a continuous variable, no significant dose–response association with overall cancer risk was observed. These findings should therefore be interpreted cautiously. Although FQs exposure was associated with increased overall cancer risk, the lack of a clear dose–response relationship argues against a simple direct pharmacological carcinogenic effect. Instead, the observed association may reflect confounding by indication, reverse causation, infection burden, healthcare utilization, or underlying ILD severity. Patients requiring FQs may have more frequent or severe infections, greater systemic inflammation, more clinical encounters, or occult malignancies initially presenting as infectious syndromes. For example, urinary tract symptoms or prostatitis may precede the diagnosis of prostate cancer, potentially explaining the strong association observed for prostate cancer [15,16,17,18,19,20,21,22,23,24,25]. The reduced risk of immune-related cancers may reflect residual confounding, differences in immune-modulating medication use, competing risks, or heterogeneous biological pathways rather than a definitive protective effect. Because claims data do not contain detailed immune profiles, microbiome data, inflammatory biomarkers, smoking intensity, pulmonary function, imaging severity, or histopathological ILD subtype, mechanistic conclusions cannot be drawn [18,19,20,21,22,23,24,25,26]. Overall, our findings indicate that FQs exposure in ILD patients is associated with increased overall cancer risk after correction for immortal time bias, but the absence of a consistent dose–response pattern suggests that this association is more likely influenced by clinical indication and underlying disease complexity than by a direct causal drug effect.

### 3.2. Strengths

This study has several strengths. First, we used a large nationwide claims-based database with long-term follow-up, allowing assessment of overall and site-specific cancer outcomes in a clinically vulnerable ILD population. Second, propensity score matching was applied to balance baseline demographic characteristics, comorbidities, and concomitant medications between FQ users and non-users. Third, immortal time bias was specifically addressed by defining the index date for FQ users as the first FQ prescription after ILD diagnosis and assigning comparable randomized index dates to non-users. Fourth, multiple statistical approaches, including standard Cox regression, time-dependent Cox regression, and Fine–Gray competing-risk models, were used to test the robustness of the findings. Finally, both cumulative duration and cumulative dose analyses were performed, helping to evaluate whether the observed association followed a biologically plausible dose–response pattern.

### 3.3. Limitations

Several limitations should be acknowledged. First, this was an observational study; therefore, causal inference cannot be established. Although propensity score matching and multivariable adjustment were performed, residual confounding remains possible. Second, confounding by indication is a major concern because FQs are prescribed for infections that may themselves be related to cancer risk, occult malignancy, or disease severity. Third, reverse causation cannot be excluded, particularly if early cancer manifestations were initially treated as infections before cancer diagnosis. Fourth, claims data lack important clinical information, including smoking status, alcohol use, occupational exposures, ILD severity, pulmonary function, radiological fibrosis, socioeconomic status, body mass index, environmental exposures, microbiome composition, inflammatory biomarkers, and laboratory data. Fifth, medication adherence could not be confirmed, and prescription records may not fully reflect actual drug exposure. Sixth, the lack of a clear dose–response relationship limits biological interpretation and weakens support for a direct carcinogenic effect of FQs. Finally, although competing-risk and time-dependent models were applied, unmeasured differences in mortality, healthcare-seeking behavior, cancer screening intensity, and infection burden may still have influenced the results [27,28,29,30,31,32,33].

## 4. Materials and Methods

### 4.1. Data Source

We analyzed the Longitudinal Generation Tracking Database 2000 (LGTD 2000), a representative subset of the National Health Insurance Research Database (NHIRD). The LGTD 2000 contains comprehensive demographic information, inpatient and outpatient records, and data on medications and treatments for 2 million insured individuals. All personal identification numbers were encrypted to ensure patient privacy. This study was approved by the Institutional Review Board (IRB) of the China Medical University Hospital Research Ethics Committee (CMUH112-REC1-117(CR-2)).

### 4.2. Study Population 

Initially, we identified patients with ILD from the Longitudinal Generation Tracking Database (LGTD 2000). The ILD cohort comprised patients who had ≥3 outpatient visits or ≥1 hospitalization for ILD (newly diagnosed) between 2000 and 2020. Diagnoses were identified using the International Classification of Diseases, Ninth Revision, Clinical Modification (ICD-9-CM codes, including 135, 237.7, 272.2, 277.3, 277.8, 500–505, 506.4, 508.1, 508.8, 515–516, 446.21, 446.4, 495, 517.2, 517.8, 518.3, 555, 710, 714.81, 720, and 759.5; ICD-10-CM D86, E78.2, E85, E88.89, J60-61, J62.8, J63, J64, J66, J67, J68.4, J70.1, J70.8, J82, J84, J99, M31.0, M31.3, M32, M33, M34, M35.0, M05.1, M45, K50, Q85.0, Q85.1). Full name can be found in Table S1 of Supplementary Materials.

FQs were indicated for various infections, such as complicated urinary tract infections (UTIsi)), complicated pyelonephritis, chronic bacterial prostatitis, intra-abdominal infections, and lower respiratory tract infections (including acute exacerbations of COPD and nosocomial pneumonia). Other indications included tuberculosis (TB), sinusitis, skin and soft-tissue infections (e.g., gout with bacterial infection), bone and joint infections (e.g., periodontal diseases), and febrile neutropenia (e.g., in patients with diabetes mellitus [DM]). To strictly prevent immortal time bias, we applied a rigorous index date assignment strategy. For FQ users, the index date was defined strictly as the date of their first FQ prescription following the ILD diagnosis, and follow-up commenced only from this date. Any person-time prior to this first prescription was not attributed to the exposed group. For non-users, to mimic the time-to-exposure distribution of the user cohort and ensure comparable follow-up periods, we assigned a randomized index date distributed between their ILD diagnosis and the end of the study period. Patients who developed cancer or died before their respective index dates were excluded. Those who never received FQs after the ILD diagnosis were designated as non-users. Patients aged ≤ 18 years, who had a history of cancer before participating in this study or had incomplete demographic information, were excluded. To ensure a balanced comparison, the study employed propensity score matching to select FQ users and non-users by age group, sex, and index year, resulting in a total sample of 7906 patients.

### 4.3. Main Outcome and Covariates

The primary endpoint of this study was the development of cancer (ICD-9-CM code: 140-208; ICD-10-CM C00-C99), as evidenced by a major illness or injury certificate for cancer. The study endpoint was defined as the occurrence of cancer, withdrawal from the insurance program, or 31 December 2021.

### 4.4. Selection of Comorbidities and Medications

In Taiwan, respiratory, gastrointestinal, and genitourinary syndromes are the leading causes of emergency department visits. Clinically, pneumonia, gastroenteritis, and UTI are among the most prevalent diagnoses in hospital settings [15]. It is noteworthy that certain malignancies may initially present as common infections, particularly in immunocompromised patients or those with metabolic syndrome; for instance, obstructive pneumonia may mask underlying lung cancer, UTIs may be associated with prostate cancer, and gastroenteritis may manifest in patients with colon cancer. To account for these potential confounding factors, this study included patients with cancer-associated infections—such as tuberculosis, pneumonia, peptic ulcer disease, irritable bowel syndrome, and pelvic inflammatory disease—as well as those with benign prostatic hypertrophy [15,16]. Furthermore, immunocompromised individuals and those with metabolic or chronic conditions (e.g., COPD, cirrhosis, atopic diseases, chronic kidney disease, congestive heart failure, stroke, hypertension, hyperlipidemia, and diabetes mellitus) were incorporated into the analysis. Notably, the use of anti-inflammatory medicines, or immunosuppressants, was specifically included as a key pharmacological factor in our analysis [17]. Immune-related cancers were defined as malignancies potentially associated with immune dysregulation, chronic inflammation, infection-related carcinogenesis, or impaired immune surveillance, including hematologic malignancies, infection-related cancers, inflammation-associated cancers, and cancers commonly observed under immunosuppressed conditions [14,15,16,17].

### 4.5. Statistical Analysis

Categorical variables were expressed as numbers and percentages, and differences between the two cohorts were examined using the chi-square test. Continuous variables were presented as a mean and standard deviation, and differences between the two cohorts were assessed using Student’s t-test. The incidence rate was expressed as per 1000 person-years. Cox proportional hazard models were used to estimate crude and adjusted hazard ratios (aHRs) with 95% confidence intervals (CIs) for cancer risk. The sub-classification of cancers includes hematologic, head and neck, esophagus, stomach, colon–rectum, liver, pancreas, lung, skin, breast, immune-related cancers, cervix/endometrium/ovary, prostate, bladder, kidney, brain, and thyroid were included in the analysis. To assess the robustness of our findings, we evaluated FQ exposure using two distinct metrics: the duration of therapy (days) and cumulative dose (mg). This dual approach allows for a comprehensive assessment of the dose-dependent impact of FQs on subsequent cancer risk. We also used a time-dependent Cox proportional hazards regression model and competing-risks models to assess the risk of cancer as a sensitivity analysis. Furthermore, to evaluate the dose–response relationship and address concerns regarding the arbitrary categorization of drug exposure, fluoroquinolone exposure was also modeled as a continuous variable. The cumulative duration (in days) and cumulative dose (in mg) of fluoroquinolone therapy were entered into the Cox proportional hazards regression to formally assess the linear dose–response relationship and to validate the robustness of the categorical findings. The Kaplan–Meier method was used to obtain the cumulative curves, and the results were then examined using the log-rank test. SAS statistical software (version 9.4, SAS Institute, Cary, NC, USA) was used to perform the analysis. A *p*-value was set at 0.05.

## 5. Conclusions

After correction for immortal time bias, FQ exposure was associated with an increased long-term risk of overall cancer among patients with ILD. A particularly strong association was observed for prostate cancer, whereas immune-related cancers showed a reduced risk. However, the absence of a consistent dose–response relationship suggests that these findings may be driven by confounding by indication, reverse causation, occult malignancy, or underlying ILD severity rather than a direct pharmacological carcinogenic effect. Further studies using active-comparator new-user designs, lag-time analyses, target trial emulation, and marginal structural models are warranted.

## Figures and Tables

**Table 1:** Baseline characteristics of fluoroquinolone (FQ) users and non-users (N = 3953).

	Fluoroquinolone		
Variable	No (N = 3953) N (%)	Yes (N = 3953) N (%)	*p*-Value
**Age**			0.58
≤49	607 (15.4%)	641 (16.2%)	
50–64	1205 (30.5%)	1193 (30.2%)	
65+	2141 (54.2%)	2119 (53.6%)	
Mean Age (SD) ^#^	66.1 (15.0)	65.8 (15.1)	0.43
**Sex**			0.43
Female	1934 (48.9%)	1899 (48.0%)	
Male	2019 (51.1%)	2054 (52.0%)	
**Comorbidities**			
Tuberculosis	499 (12.6%)	524 (13.3%)	0.40
Pneumonia	2503 (63.3%)	2510 (63.5%)	0.87
Peptic ulcer disease	2825 (71.5%)	2157(54.6)	0.77
COPD	2170 (54.9%)	3077 (56.7%)	0.56
Cirrhosis	515 (13.0%)	534 (13.5%)	0.53
Atopic disease	2678 (67.8%)	2661 (67.3%)	0.68
Diabetes	2244 (56.8%)	2265 (57.3%)	0.63
Hypertension	3197 (80.9%)	3187 (80.6%)	0.78
Hyperlipidemia	2997 (75.8%)	4000 (73.7%)	0.35
Chronic kidney disease	1911 (48.3%)	1853 (48.7%)	0.19
Stroke	1673 (42.3%)	1694 (42.9%)	0.63
Pelvic inflammatory disease	5 (0.13%)	8 (0.20%)	0.41
Heart failure	1247 (31.6%)	1298 (32.8%)	0.22
Periodontal diseases	3093 (78.2%)	3066 (77.8%)	0.46
IBS	893 (22.6%)	871 (22.0%)	0.55
Prostate inflammation (incl. BPH)	1414 (35.8%)	1394 (35.3%)	0.64
Anti-inflammatory medicines ^a^	3858 (97.6%)	3857 (97.6%)	0.94
Immunosuppressants ^b^	2547 (64.4%)	2493 (63.1%)	0.21

Chi-square test; ^#^ *t*-test; SD: Standard deviation. BPH: Benign Prostatic Hyperplasia. ^a^ Includes aspirin, statins, NSAIDs, ACE inhibitors, ARBs, CCBs, beta-blockers, diuretics. ^b^ Includes ICS, prednisolone, sulfasalazine, cyclophosphamide, methotrexate, hydroxychloroquine, and TNF-α antagonists.

**Table 2:** Risk of all-cause cancer by fluoroquinolone use.

Fluoroquinolone	N	Person-Years	Follow-Up (Mean, SD) ^a^	Events	Rate/1000 PY ^b^	Crude HR (95% CI)	Adjusted HR (95% CI) ^c^
No use	3953	23,504	5.95 (4.29)	189	8.04	1.00 (Reference)	1.00 (Reference)
Use	3953	21,462	5.43 (4.22)	247	11.5	1.45 (1.20, 1.75) ***	1.45 (1.20, 1.76) ***

^a^ SD: Standard deviation. ^b^ PY: Person-years. ^c^ Adjusted for age, sex, comorbidities, and medication use. *** *p* < 0.001.

**Table 3:** Adjusted risk of site-specific cancer by fluoroquinolone use among patients with interstitial lung disease.

	Fluoroquinolone				Fluoroquinolone Compared to Non-Fluoroquinolone	
	No		Yes		Crude HR	Adjusted HR
Cancer Site	Events	Rate ^a^	Events	Rate ^a^	(95% CI)	(95% CI) ^b^
Hematologic malignancy	10	0.43	17	0.79	1.85 (0.85, 4.04)	1.93 (0.88, 4.23)
Head and neck cancer	8	0.34	14	0.65	1.94 (0.81, 4.63)	1.66 (0.68, 4.01)
Esophagus	10	0.43	6	0.28	0.65 (0.24, 1.80)	0.61 (0.22, 1.74)
Stomach	7	0.3	12	0.56	1.94 (0.76, 4.92)	2.14 (0.82, 5.62)
Colon, rectum	20	0.85	26	1.21	1.44 (0.80, 2.58)	1.37 (0.76, 2.46)
Liver	30	1.28	26	1.21	0.97 (0.57, 1.64)	1.03 (0.61, 1.76)
Pancreas	6	0.26	4	0.19	0.75 (0.21, 2.65)	0.77 (0.21, 2.84)
Lung	34	1.45	34	1.58	1.12 (0.70, 1.80)	1.22 (0.75, 1.97)
Skin	5	0.21	6	0.28	1.32 (0.40, 4.32)	1.16 (0.34, 4.01)
Breast cancer	16	0.68	21	0.98	1.46 (0.76, 2.80)	1.38 (0.71, 2.66)
Immune-related cancers	92	1.38	57	0.85	0.62 (0.45, 0.87) **	0.62 (0.44, 0.87) **
Cervix/Endometrium/Ovary	6	0.26	6	0.28	1.12 (0.36, 3.46)	1.23 (0.39, 3.88)
Prostate	6	0.26	30	1.40	5.52 (2.30, 13.26) ***	5.50 (2.25, 13.48) ***
Bladder, kidney	12	0.51	22	1.03	2.04 (1.01, 4.13) *	2.03 (0.99, 4.14)
Brain		0.09		0.09	1.11 (0.16, 7.85)	1.36 (0.16, 11.70)
Thyroid	4	0.17	5	0.23	1.41 (0.38, 5.25)	2.12 (0.48, 9.23)
Others	40	1.7	36	1.68	1.00 (0.64, 1.57)	1.05 (0.67, 1.66)

^a^ Incidence rate per 1000 person-years ^b^ Adjusted for age, sex, comorbidities, and medications. * *p* < 0.05; ** *p* < 0.01; *** *p* < 0.001.

**Table 4:** Incidence and adjusted hazard ratio of sub-cancer stratified by cumulative use days per year of fluoroquinolone therapy in patients with interstitial lung disease-virus infection among the propensity score-matched cohort.

Medication Exposed	Event	Person-Year	Rate ^a^	Adjusted HR (95% CI) ^b^
**Colon, rectum**				
Non-fluoroquinolone	20	23,504	0.85	1.00
Fluoroquinolone ^#^				
≤35 days	14	10,141	1.38	1.53 (0.76, 3.06)
36–55 days	7	5447	1.29	1.43 (0.60, 3.41)
>56 days	5	5874	0.85	1.01 (0.37, 2.72)
**Liver**				
Non-fluoroquinolone	30	23,504	1.28	1.00
Fluoroquinolone ^#^				
≤35 days	13	10,141	1.28	1.12 (0.58, 2.17)
36–55 days	5	5447	0.92	0.76 (0.30, 1.98)
>56 days	8	5874	1.36	1.15 (0.52, 2.54)
**Lung**				
Non-fluoroquinolone	34	23,504	1.45	1.00
Fluoroquinolone ^#^				
≤35 days	13	10,141	1.28	1.05 (0.55, 2.02)
36–55 days	9	5447	1.65	1.26 (0.60, 2.64)
>56 days	12	5874	2.04	1.42 (0.72, 2.77)
**Immune-related cancers**				
Non-fluoroquinolone	40	23,504	1.7	1.00
Fluoroquinolone ^#^				
≤35 days	19	10,141	1.87	1.18 (0.68, 2.06)
36–55 days	8	5447	1.47	0.91 (0.42, 1.95)
>56 days	9	5874	1.53	0.98 (0.47, 2.03)

^a^ Incidence rate per 1000 person-years ^b^ Adjusted HR: multivariable analysis including age, sex, comorbidities, and medications. ^#^ The cumulative use days are partitioned into 3 segments by the median and the third quartile.

**Table 5:** Incidence and adjusted hazard ratio of sub-cancer stratified by cumulative use dose per year of fluoroquinolone therapy in patients with interstitial lung disease-virus infection in the propensity score-matched cohort.

Medication Exposed	Event	Person-Year	Rate ^a^	Adjusted HR (95% CI) ^b^
**Colon, rectum**				
Non-fluoroquinolone	20	23,504	0.85	1.00
Fluoroquinolone ^#^				
≤3000 mg	20	14,635	1.37	1.57 (0.84, 2.94)
3001–6000 mg	6		0.57	0.60 (0.14, 2.61)
>6000 mg			1.2	1.35 (0.45, 4.05)
**Liver**				
Non-fluoroquinolone	30	23,504	1.28	1.00
Fluoroquinolone ^#^				
≤3000 mg	18	14,635	1.23	1.04 (0.58, 1.90)
3001–6000 mg	3	3491	0.86	0.73 (0.22, 2.43)
>6000 mg	5	3337	1.5	1.31 (0.49, 3.46)
**Lung**				
Non-fluoroquinolone	34	23,504	1.45	1.00
Fluoroquinolone ^#^				
≤3000 mg	22	14,635	1.5	1.13 (0.65, 1.95)
3001–6000 mg	7	3491	2.01	1.61 (0.70, 3.67)
>6000 mg	5	3337	1.5	1.22 (0.46, 3.18)
**Immune-related cancers**				
Non-fluoroquinolone	40	23,504	1.7	1.00
Fluoroquinolone ^#^				
≤3000 mg	24	14,635	1.64	1.01 (0.61, 1.70)
3001–6000 mg	4	3491	1.15	0.73 (0.26, 2.05)
>6000 mg	8	3337	2.4	1.60 (0.73, 3.49)

^a^ Incidence rate per 1000 person-years ^b^ Adjusted HR: multivariable analysis including age, sex, comorbidities, and medications; ^#^ The cumulative use days are partitioned into 3 segments by median, and third quartile.

**Table 6:** Risk of all-cause cancer by fluoroquinolone use (time-dependent Cox regression model).

Variable	Fluoroquinolone Compared to Non-Fluoroquinolone	
	Crude HR	Adjusted HR
Cancer Site	(95% CI)	(95% CI) ^a^
Cancer	1.99 (1.52, 2.61) ***	2.07 (1.57, 2.72) ***
Hematologic malignancy	3.68 (1.40, 9.68) **	4.09 (1.51, 11.1) **
Head and neck cancer	-	-
Esophagus	0.52 (0.07, 3.99)	0.46 (0.06, 3.61)
Stomach	1.90 (0.42, 8.61)	2.36 (0.50, 11.1)
Colon, rectum	0.74 (0.23, 2.42)	0.77 (0.23, 2.55)
Liver	1.72 (0.76, 3.87)	1.71 (0.75, 3.88)
Pancreas	-	-
Lung	3.56 (2.00, 6.33) ***	3.35 (1.87, 6.01) ***
Skin	-	-
Breast cancer	0.64 (0.15, 2.68)	0.70 (0.16, 2.97)
Immune-related cancers	1.22 (0.55, 2.68)	1.21 (0.55, 2.70)
Cervix/Endometrium/Ovary	1.23 (0.15, 9.96)	1.42 (0.17, 11.7)
Prostate	8.89 (4.36, 18.2) ***	9.95 (4.75, 20.8) ***
Bladder, kidney	3.39 (1.43, 8.04) **	3.72 (1.55, 8.93) **
Brain	-	-
Thyroid	-	-
Others	1.31 (0.51, 3.35)	1.37 (0.53, 3.53)

^a^ Adjusted for age, sex, comorbidities, and medication use. ** *p* < 0.01. *** *p* < 0.001.

**Table 7:** Risk of all-cause cancer by fluoroquinolone use (competing risk model).

Variable	Fluoroquinolone Compared to Non-Fluoroquinolone	
	Crude SHR	Adjusted SHR
Cancer Site	(95% CI)	(95% CI) ^a^
Cancer	1.31 (1.08, 1.58) **	1.28 (1.06, 1.55) **
Colon, rectum	1.31 (0.73, 2.34)	1.24 (0.69, 2.22)
Liver	0.87 (0.51, 1.47)	0.88 (0.53, 1.47)
Lung	1.00 (0.62, 1.62)	1.04 (0.65, 1.68)
Immune-related cancers	0.90 (0.58, 1.42)	0.91 (0.59, 1.42)

^a^ Adjusted for age, sex, comorbidities, and medication use. ** *p* < 0.01.

**Table 8:** Hazard ratios and 95% confidence intervals for all-cause cancer risk by continuous dose of fluoroquinolones.

Variable	Fluoroquinolone Compared to Non-Fluoroquinolone	
	Crude HR	Adjusted HR
Cancer Site	(95% CI)	(95% CI) ^a^
Cancer	0.99 (0.99, 1.00)	0.99 (0.99, 1.00)

^a^ Adjusted for age, sex, comorbidities, and medication use.

## Data Availability

The dataset is held by the Taiwan Ministry of Health and Welfare and is available upon request.

## Supplementary Materials

The following supporting information can be downloaded at: https://www.mdpi.com/article/10.3390/ph19071067/s1, Table S1. Full List of ICD-9-CM and ICD-10-CM Codes.

## Abbreviations

FQs: Fluoroquinolones; ILD: Interstitial Lung Disease; ICD-9-CM: International Classification of Diseases, Ninth Revision, Clinical Modification; NHIRD: National Health Insurance Research Database; aHRs: Adjusted Hazard Ratios; CIs: Confidence Intervals; COPD: Chronic Obstructive Pulmonary Disease; TB: Tuberculosis; DDD: Defined Daily Dose.
