# Supplementary material for: Fluoroquinolone Exposure and Cancer Risk in Interstitial Lung Disease: A Propensity-Score-Matched Cohort Study Using Cox and Competing-Risk Models
Source: Pharmaceuticals (Basel). 2026 Jul 10;19(7):1067. doi: 10.3390/ph19071067 (PMC13416382; doi:10.3390/ph19071067)
Supplement: Supplementary file 1 [file pharmaceuticals-19-01067-s001.zip › pharmaceuticals-4327074-supplementary.pdf]

Table S1. Full List of ICD-9-CM and ICD-10-CM Codes.

ICD-9-CM Code	ICD-9-CM Diagnosis	ICD-10-CM Code(s)	ICD-10-CM Diagnosis
135	Sarcoidosis	D86	Sarcoidosis
237.7	Neurofibromatosis	Q85.0	Neurofibromatosis, type 1
272.2	Mixed hyperlipidemia	E78.2	Mixed hyperlipidemia
277.3	Amyloidosis	E85	Amyloidosis
277.8	Other specified metabolic disorders	E88.89	Other specified metabolic disorders
500	Coal workers' pneumoconiosis	J60	Coal workers' pneumoconiosis
501	Asbestosis	J61	Pneumoconiosis due to asbestos and other mineral fibers
502	Pneumoconiosis due to silica or silicates	J62.8	Pneumoconiosis due to other dust containing silica
503	Pneumoconiosis due to other inorganic dust	J63	Pneumoconiosis due to other inorganic dusts
504	Pneumopathy due to inhalation of other dust	J66	Airway disease due to specific organic dust
505	Pneumoconiosis, unspecified	J64	Unspecified pneumoconiosis
506.4	Chronic respiratory conditions due to fumes and vapors	J68.4	Chronic respiratory conditions due to chemicals, gases, fumes, and vapors
508.1	Pulmonary manifestations due to radiation	J70.1	Chronic and other pulmonary manifestations due to radiation
508.8	Other respiratory conditions due to external agents	J70.8	Other respiratory conditions due to other external agents
515	Postinflammatory pulmonary fibrosis	J84	Other interstitial pulmonary diseases
516	Other alveolar and parietoalveolar pneumonopathy	J84	Other interstitial pulmonary diseases
446.21	Goodpasture's syndrome	M31.0	Hypersensitivity angiitis
446.4	Wegener's granulomatosis	M31.3	Granulomatosis with polyangiitis
495	Extrinsic allergic alveolitis	J67	Hypersensitivity pneumonitis due to organic dust
517.2	Lung involvement in systemic sclerosis	M34 + J99	Systemic sclerosis with respiratory involvement
517.8	Lung involvement in other diseases	J99	Respiratory disorders in diseases classified elsewhere
518.3	Pulmonary eosinophilia	J82	Pulmonary eosinophilia
555	Regional enteritis (Crohn's disease)	K50	Crohn's disease
710	Diffuse connective tissue disease	M35.9	Systemic involvement of connective tissue, unspecified
714.81	Rheumatoid lung	M05.1	Rheumatoid lung disease with rheumatoid arthritis
720	Ankylosing spondylitis	M45	Ankylosing spondylitis
759.5	Tuberous sclerosis	Q85.1	Tuberous sclerosis
